# Supplementary material for: Calibrated muscle models improve tracking simulations without enhancing gait predictions
Source: PLoS One. 2025 Jul 1;20(7):e0327172. doi: 10.1371/journal.pone.0327172 (PMC12212882; doi:10.1371/journal.pone.0327172)
Supplement: S1 File — A PDF file including some details regarding the optimal control problem for the gait predictive simulations and additional figures (PDF) [file pone.0327172.s001.pdf]

Supplementary Material for: Calibrated Muscle Models Improve Tracking Simulations Without Enhancing Gait Predictions

Filippo Maceratesi, Míriam Febrer-Nafría, and Josep M. Font-Llagunes

PLOS ONE

Optimal control problem for the gait predictive simulations

Design variables

<u>States</u>:

- joint coordinates (q),
- joint velocities ($\dot{q}$),
- muscle activations (a),
- normalized tendon forces ($\widetilde{F^T}$).

<u>Controls</u>:

- joint accelerations ($\ddot{q}$),
- time derivative of muscle activations (u_a),
- ground reaction loads (u_{GRF}),
- time derivative of the normalized tendon forces (u_f).

Cost function

The following cost function J was used in the optimal control problem (OCP):

$$J = \frac{1}{d}\int_{t_0}^{t_f} (w_1(\dot{E})^2 + w_2(a)^2 + w_3(\ddot{q})^2 + w_4(T_p)^2)dt \qquad (1)$$

where d is the anteroposterior distance travelled by the pelvis in the predicted gait, $\dot{E}$ is the metabolic energy rate calculated using the phenomenological model described by Bhargava *et al.* [1], w_{1-5} are the weight factors attributed to each cost function term and T_p is the passive joint torques calculated through the passive musculotendon forces. The following weight factors were used: $w_1 = 0.1, w_2 = 0.1, w_3 = 0.001, w_4 = 0.0001$.

Path constraints

The following path constraints were included in the problem formulation:

$$-1 \leq R \leq 1 \qquad (2)$$

$$-8 \leq T^{ID} - T^M \leq 8 \qquad (3)$$

$$-1 \leq F^M \cos\alpha^M - F^T \leq 1 \qquad (4)$$

$$u_a + \frac{a}{\tau_d} \geq 0 \qquad (5)$$

$$u_a + \frac{a}{\tau_a} \leq \frac{1}{\tau_a} \tag{6}$$

$$0.1 \leq \widetilde{l^M} \leq 2.0 \tag{7}$$

$$-0.1 \leq v_{calcn,x} \leq 0.1 \tag{8}$$

$$-0.4 \leq v_{toes,x} \leq 0.4 \tag{9}$$

$$-5 \leq u_{GRF} - GRF_{CM} \leq 5 \tag{10}$$

$$-0.07 \leq d_{F-P,z} \leq 0.15 \tag{11}$$

$$0.003 \leq d_{feet,z} \leq 0.2 \tag{12}$$

$$0 \leq d_{P-T,z} \leq 0.08 \tag{13}$$

where R are the residual forces and torques at the pelvis, T^{ID} are the lower body joint torques calculated through inverse dynamics at each iteration of the OCP, T^M are the joint torques resulting from the musculotendon forces, F^M are the muscle forces, α^M are the muscle pennation angles, F^T are the tendon forces, $\tau_d = 60$ ms is the deactivation time constant, $\tau_a = 15$ ms is the activation time constant, $\widetilde{l^M}$ are the normalized muscle lengths, $v_{calcn,x}$ are the anteroposterior velocities of the calcaneus bodies of the OpenSim model during stance, $v_{toes,x}$ are the anteroposterior velocities of the toes bodies of the OpenSim model during stance, GRF_{CM} are the ground reaction loads computed through the foot-ground contact model described in [2], $d_{F-P,z}$ are the mediolateral distances between the center of the pelvis and the centers of the feet, $d_{feet,z}$ is the mediolateral distance between the centers of the two feet and, $d_{P-T,z}$ is the mediolateral distance between the center of the torso and the center of the pelvis. Constraints (2) and (3) were used to ensure dynamic consistency. Constraint (4) was used to implicitly model the contraction dynamics, whereas (5) and (6) were employed to implicitly implement the activation dynamics. Constraint (7) bounded the normalized muscle length to ensure that the muscles worked within feasible operating conditions. Constraints (8) and (9) ensured that the feet did not move or slide in the anteroposterior direction during stance. Constraint (10) was added to make sure that the ground reaction loads in the controls of the OCP were close to the ground reaction loads estimated through the foot-ground contact model. This constraint was added to facilitate the convergence of the problem. The last three constraints were included to avoid unnatural movements during gait: (11) limited excessive circumduction during gait, (12) ensured that a plausible step width was predicted and (13) prevented exaggerated mediolateral movement of the upper body.

Dynamic constraints

Since the problem dynamics were defined implicitly [3], the dynamic constraints were simply the time derivatives of the states:

$$\frac{dq}{dt} = \dot{q} \tag{14}$$

$$\frac{d\dot{q}}{dt} = \ddot{q} \tag{15}$$

$$\frac{da}{dt} = u_a \tag{16}$$

$$\frac{d\widetilde{F^T}}{dt} = u_f \tag{17}$$

Event constraints

An event constraint was included to bound the stride length of the predicted gait within some tolerance:

$$-0.05 \leq \left(x_{RF,t_f} - x_{RF,t_0}\right) - SL_{exp} \leq 0.05 \tag{18}$$

where SL_{exp} is the average stride length achieved during the gait motion capture experiments and, x_{RF,t_f} and x_{RF,t_0} are the anteroposterior positions of the right foot at the last and initial timestep of the predicted gait cycle, respectively.

Initial guess

Initial guess for the states:

- q: experimental joint coordinates from one of the recorded gait cycles,
- $\dot{q}$: spline-based time derivatives of the experimental joint coordinates,
- a: a constant activation of 0.3,
- $\widetilde{F^T}$: the above guess was input into the contraction dynamics model of the Hill-type actuators to compute a dynamically consistent guess for $\widetilde{F^T}$.

Initial guess for the controls:

- $\ddot{q}$: spline-based second time derivatives of the experimental joint coordinates,
- u_a: spline-based time derivative of the initial guess of a,
- u_{GRF}: experimental ground reaction loads from on the recorded gait cycles,
- u_f: spline-based time derivative of the initial guess of $\widetilde{F^T}$.

Supplementary figures

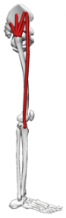

SUPERIOR GLUTEAL NERVE	
**Muscle**	**OpenSim**
**Gluteus medius**	**glmed1**
	**glmed2**
	**glmed3**
**Gluteus minimus**	**glmin1**
	**glmin2**
	**glmin3**
Tensor fasciae latae	tfl

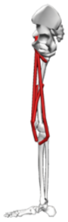

FEMORAL NERVE	
**Muscle**	**OpenSim**
Iliacus	iliacus
**Rectus femoris**	**recfem**
Sartorius	sart
**Vastus intermedius**	**vasint**
**Vastus lateralis**	**vaslat**
**Vastus medialis**	**vasmed**

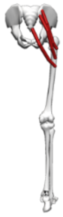

INFERIOR GLUTEAL NERVE	
**Muscle**	**OpenSim**
Gluteus maximum	glmax1
	glmax2
	glmax3

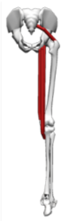

SCIATIC NERVE	
**Muscle**	**OpenSim**
Adductor magnus (ischiocondylar part)	addmagIsch
Piriformis	piri
**Semimembranosus**	**semimem**
**Semitendinosus**	**semiten**

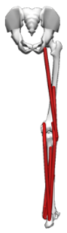

TIBIAL NERVE	
**Muscle**	**OpenSim**
**Biceps femoris longhead/shorthead**	**bflh**
	**bfsh**
Flexor digitorum longus	fdl
Flexor hallucis longus	fhl
**Gastrocnemius lateralis**	**gaslat**
**Gastrocnemius medialis**	**gasmed**
**Soleus**	**soleus**
Tibialis posterior	tibpost

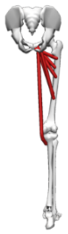

OBTURAL NERVE	
**Muscle**	**OpenSim**
Adductor brevis	addbrev
Adductor longus	addlong
Adductor magnus (adductor part)	addmagDist
	addmagMid
	addmagProx
Gracilis	grac

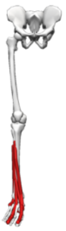

DEEP FIBULAR NERVE	
**Muscle**	**OpenSim**
Extensor digitorum longus	edl
Extensor hallucis longus	ehl
**Tibialis anterior**	**tibant**
**FIBULAR NERVE**	
**Muscle**	**OpenSim**
Peroneus brevis	perbrev
Peroneus longus	perlong

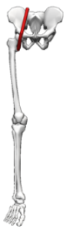

LUMBAR PLEXUS NERVE	
**Muscle**	**OpenSim**
Psoas	psoas

Fig S1. The 40 muscles included in one leg of the musculoskeletal model and their respective abbreviations in OpenSim. The muscles that were calibrated are highlighted in bold, and those that were measured with the EMG device are underlined. The muscles sharing the same innervation are grouped together in a table. Within a table, if a measured muscle shares the same main actuation contribution with other muscles, they are sub-grouped together and colored in blue. These subgroups of muscles are assumed to have the same EMG profiles.

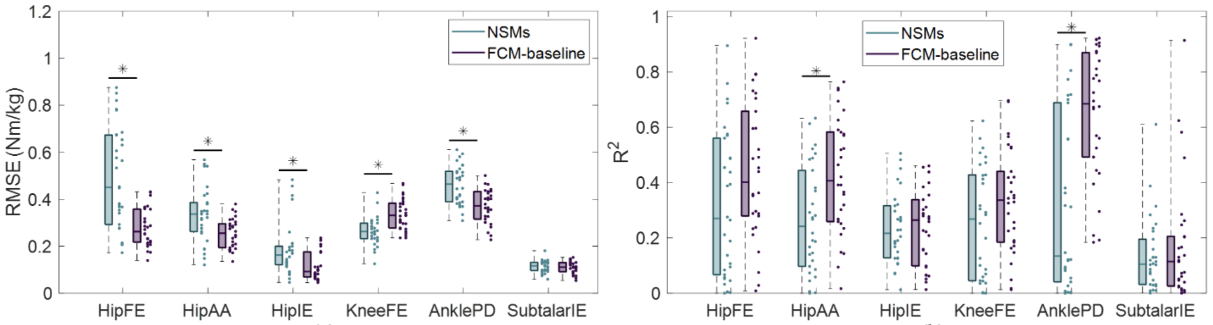

(a) (b)

Fig S2: (a) Box plots of the RMSEs divided by body mass between the ID and estimated joint torques, when using FCM-baseline and NSMs in gait tracking simulations. The dots on the right-hand side of each box plot indicate the individual RMSE values for the right and left legs of each subject. An asterisk is shown if the difference between the RMSEs of NSMs and FCM-baseline is statistically significant. (b) Box plots of the R^2 values between the ID and estimated joint torques, when using FCM-baseline and NSMs in gait tracking simulations. The dots on the right-hand side of each box plot indicate the individual R^2 values for the right and left legs of each subject. An asterisk is shown if the difference between the R^2 of NSMs and FCM-baseline is statistically significant.

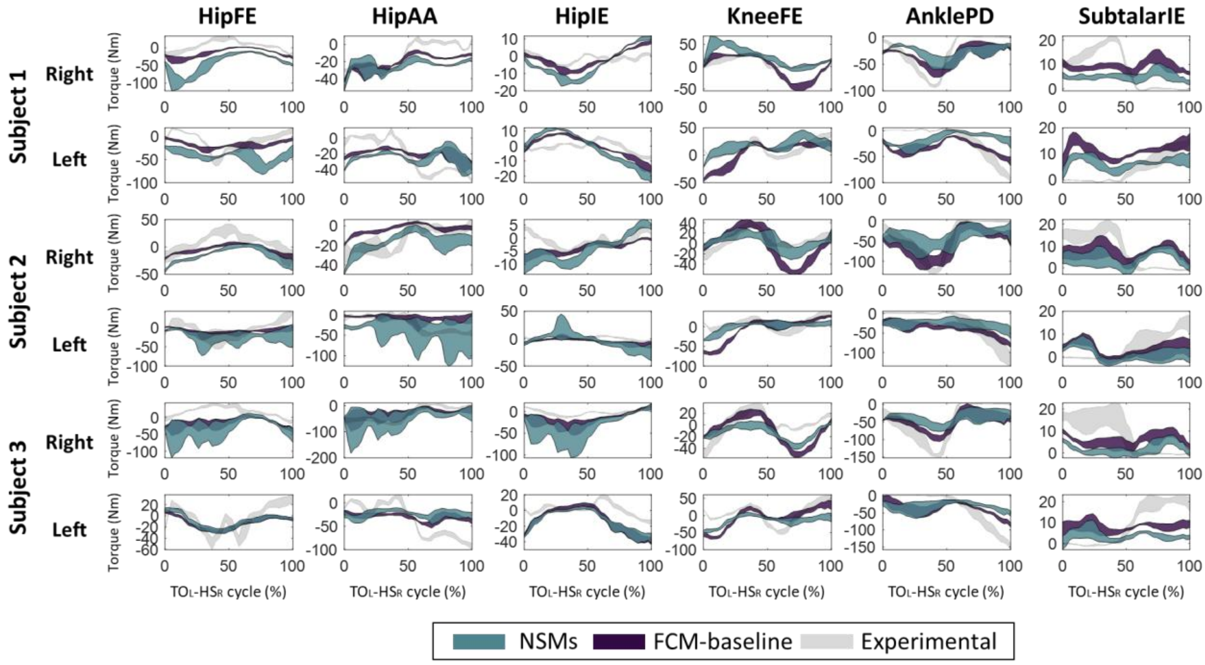

Fig S3: The standard deviations of the hipFE, hipAA, hipIE, kneeFE, anklePD and subtalarIE torque profiles of NSMs and FCM-baseline in the gait tracking simulations, compared to the respective experimental torques for the three subjects. The torque profiles are shown from left toe-off (TO_L) to right heel-strike (HS_R), corresponding to the period when the subjects are in contact with the force plates.

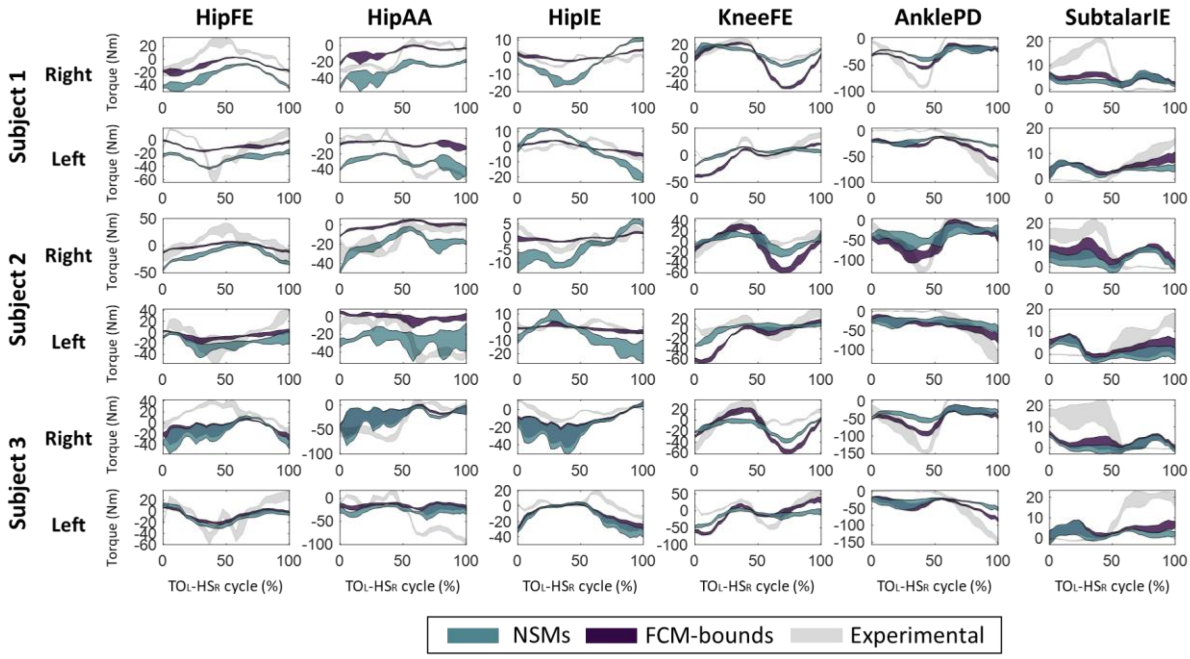

Fig S4: The standard deviations of the hipFE, hipAA, hipIE, kneeFE, anklePD and subtalarIE torque profiles of NSMs and FCM-bounds in the gait tracking simulations, compared to the respective experimental torques for the three subjects. The torque profiles are shown from left toe-off (TO_L) to right heel-strike (HS_R), corresponding to the period when the subjects are in contact with the force plates.

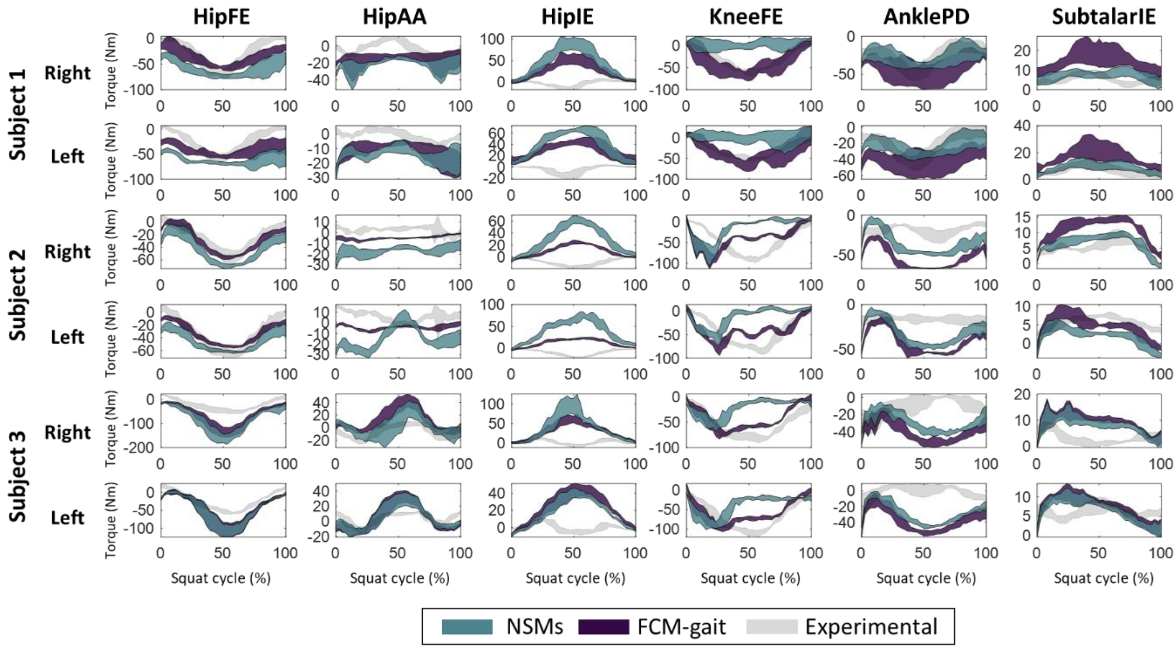

Fig S5: The standard deviations of the hipFE, hipAA, hipIE, kneeFE, anklePD and subtalarIE torque profiles of NSMs and FCM-gait in the squat tracking simulations, compared to the respective experimental torques for the three subjects.

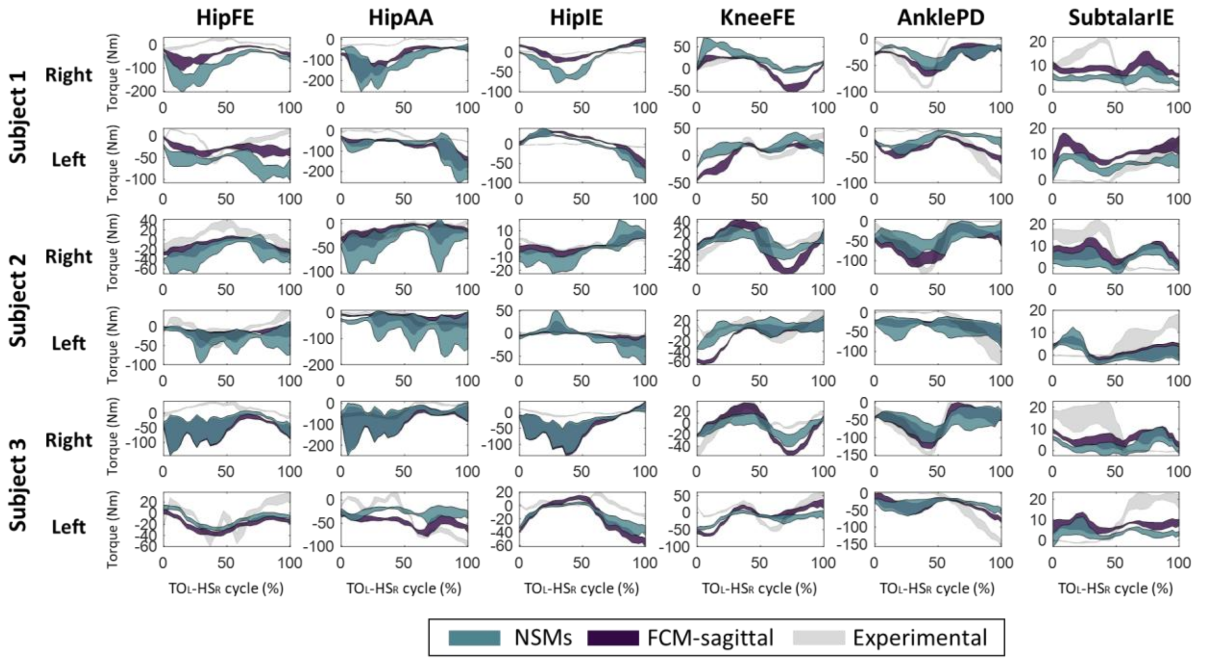

Fig S6: The standard deviations of the hipFE, hipAA, hipIE, kneeFE, anklePD and subtalarIE torque profiles of NSMs and FCM-sagittal in the gait tracking simulations, compared to the respective experimental torques for the three subjects. The torque profiles are shown from left toe-off (TO_L) to right heel-strike (HS_R), corresponding to the period when the subjects are in contact with the force plates.

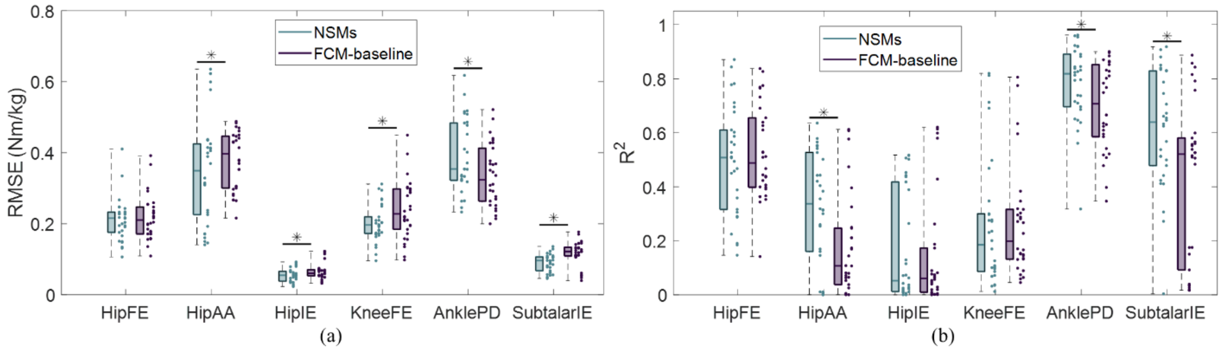

Fig S7: (a) Box plots of the RMSEs divided by body mass between the ID and estimated joint torques, when using FCM-baseline and NSMs in gait predictive simulations. The dots on the right-hand side of each box plot indicate the individual RMSE values for the right and left legs of each subject. An asterisk is shown if the difference between the RMSEs of NSMs and FCM-baseline is statistically significant. (b) Box plots of the R^2 values between the ID and estimated joint torques, when using FCM-baseline and NSMs in gait predictive simulations. The dots on the right-hand side of each box plot indicate the individual R^2 values for the right and left legs of each subject. An asterisk is shown if the difference between the R^2 of NSMs and FCM-baseline is statistically significant.

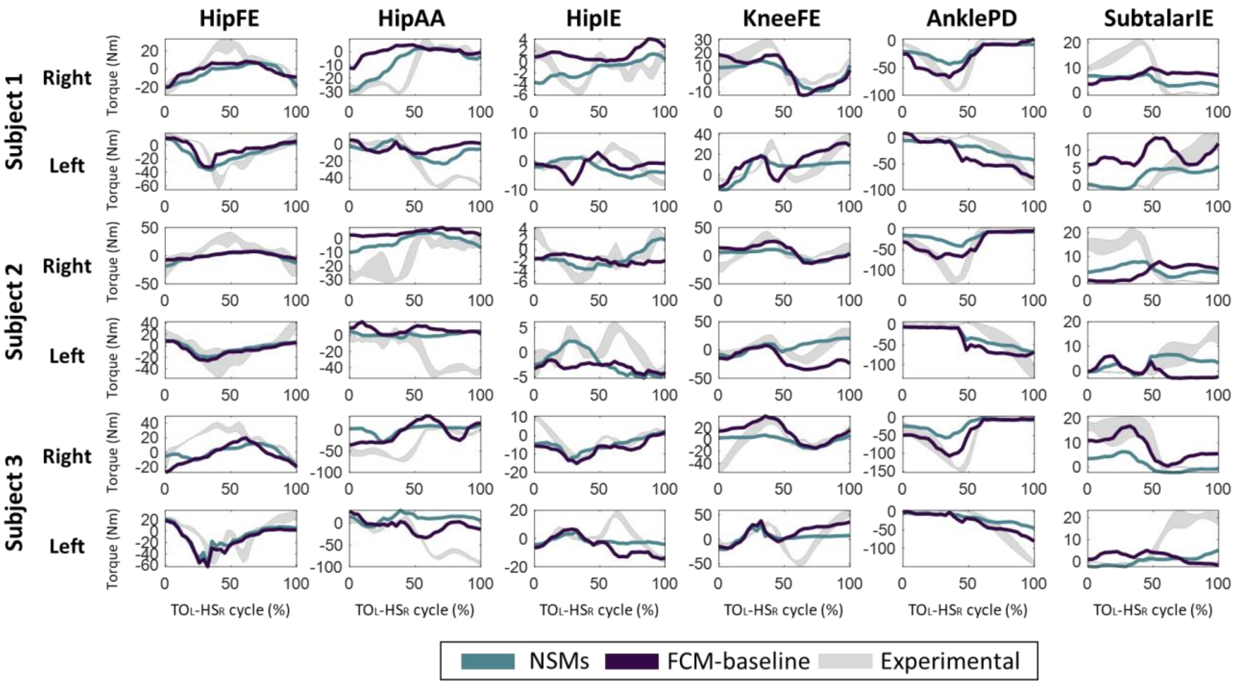

Fig S8: The mean hipFE, hipAA, hipIE, kneeFE, anklePD and subtalarIE torque profiles of NSMs and FCM-baseline in gait predictive simulations, compared to the respective experimental torques for the three subjects. The torque profiles are shown from left toe-off (TO_L) to right heel-strike (HS_R), corresponding to the period when the subjects are in contact with the force plates.

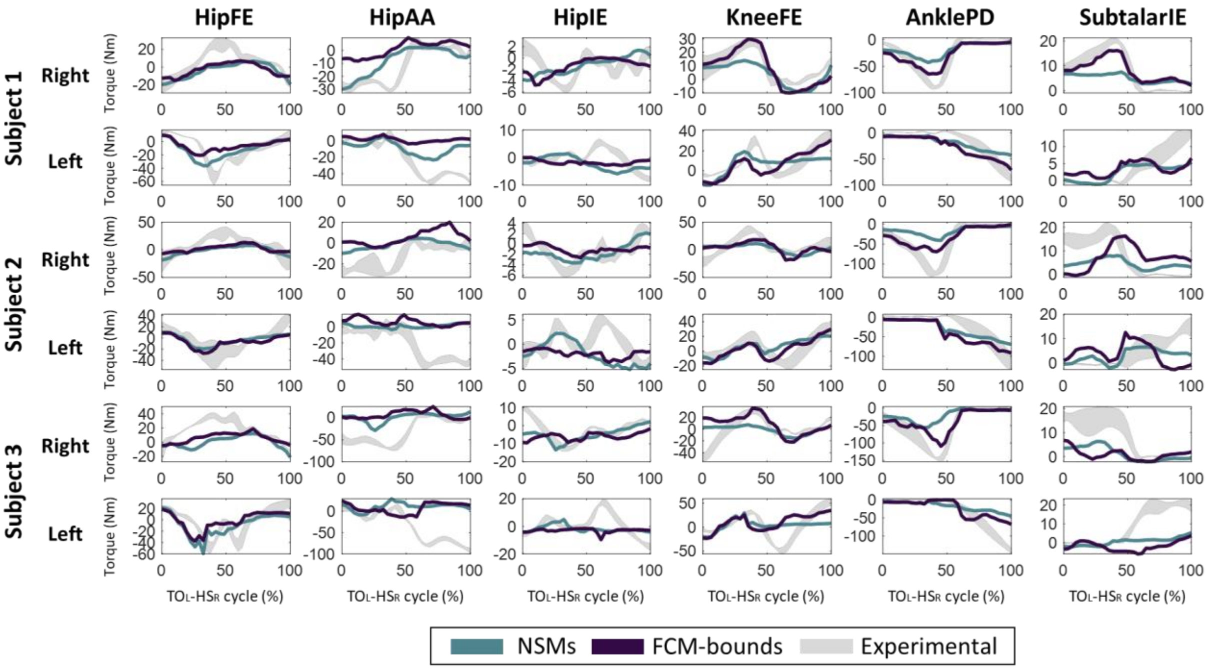

Fig S9: The mean hipFE, hipAA, hipIE, kneeFE, anklePD and subtalarIE torque profiles of NSMs and FCM-bounds in gait predictive simulations, compared to the respective experimental torques for the three subjects. The torque profiles are shown from left toe-off (TO_L) to right heel-strike (HS_R), corresponding to the period when the subjects are in contact with the force plates.

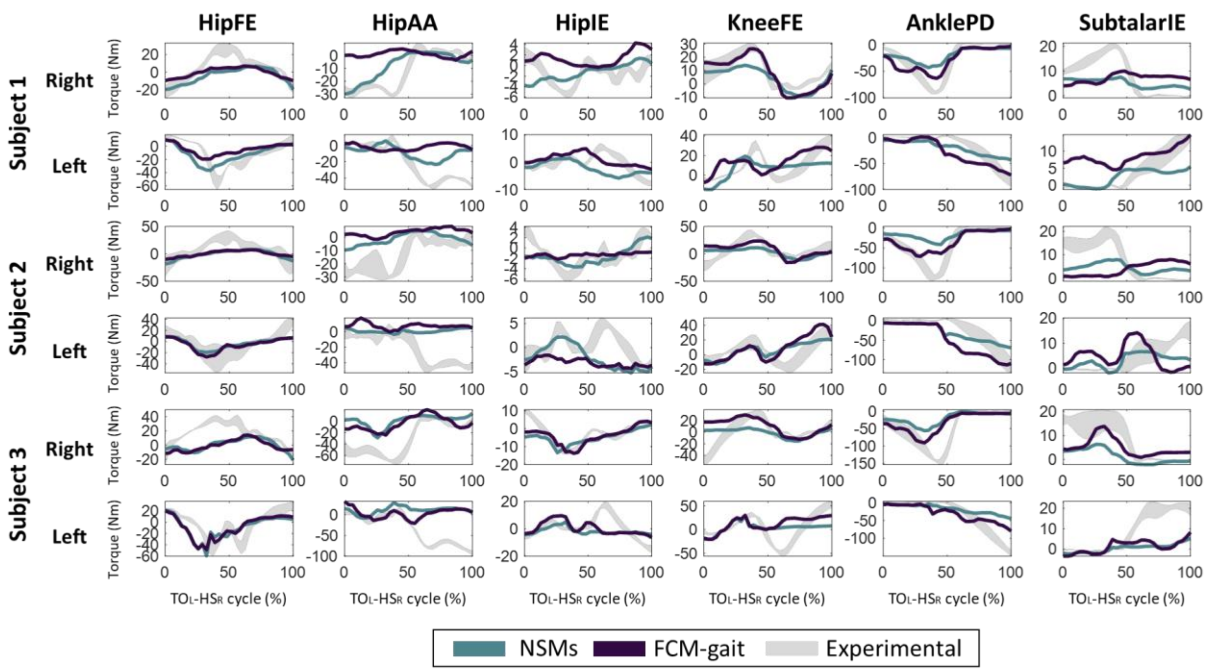

Fig S10: The mean hipFE, hipAA, hipIE, kneeFE, anklePD and subtalarIE torque profiles of NSMs and FCM-gait in gait predictive simulations, compared to the respective experimental torques for the three subjects. The torque profiles are shown from left toe-off (TO_L) to right heel-strike (HS_R), corresponding to the period when the subjects are in contact with the force plates.

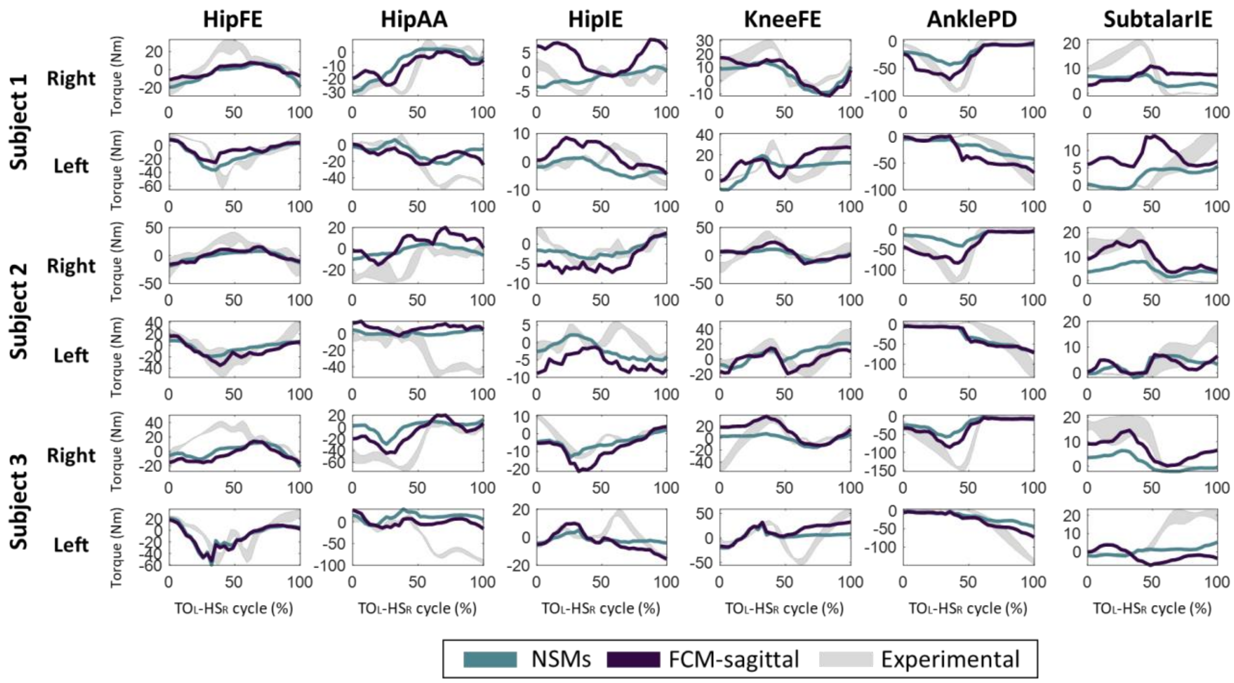

Fig S11: The mean hipFE, hipAA, hipIE, kneeFE, anklePD and subtalarIE torque profiles of NSMs and FCM-sagittal in gait predictive simulations, compared to the respective experimental torques for the three subjects. The torque profiles are shown from left toe-off (TO_L) to right heel-strike (HS_R), corresponding to the period when the subjects are in contact with the force plates.

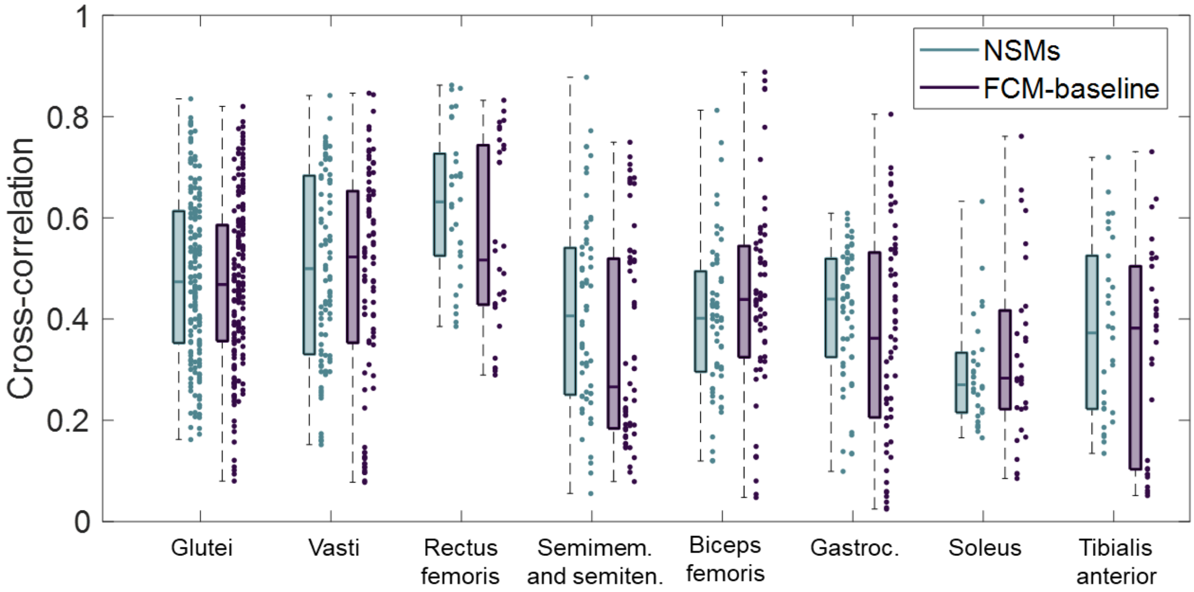

Fig S12. Box plots of the cross-correlation between the EMG signals and excitations of the MT models, when using FCM-baseline and NSMs in gait predictive simulations. The dots on the right-hand side of each box plot indicate the individual cross-correlation values for the right and left legs of each subject.

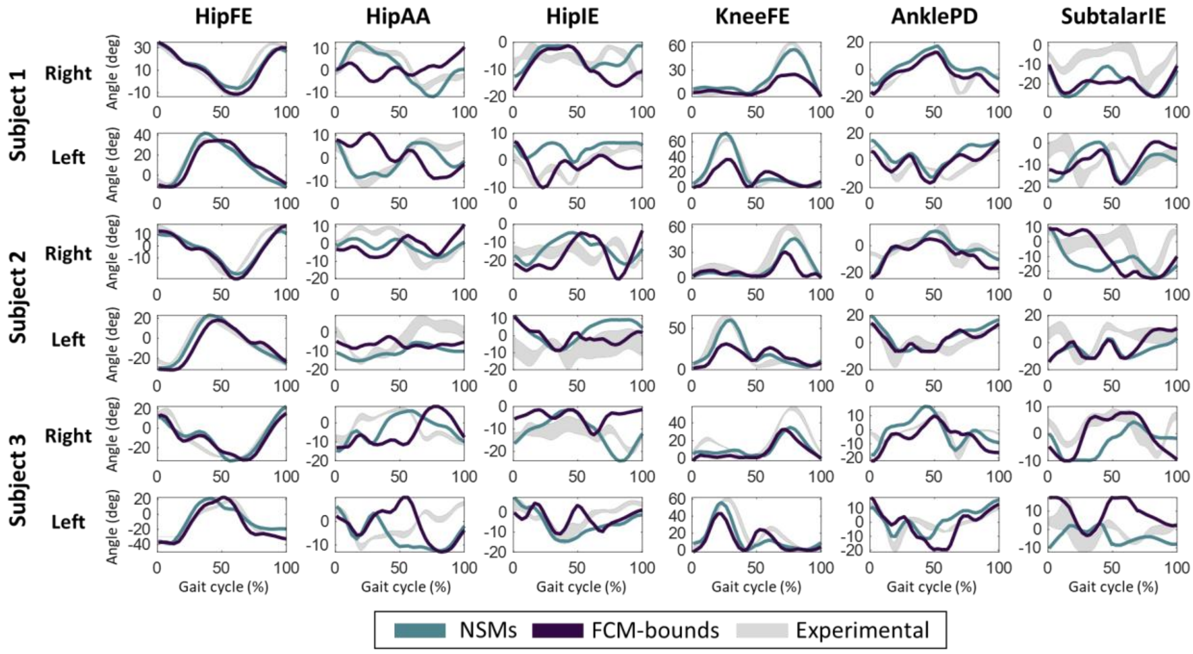

Fig S13. The predicted hipFE, hipAA, hipIE, kneeFE, anklePD and subtalarIE angles of NSMs and FCM-bounds, compared to the respective experimental joint angles for the three subjects.

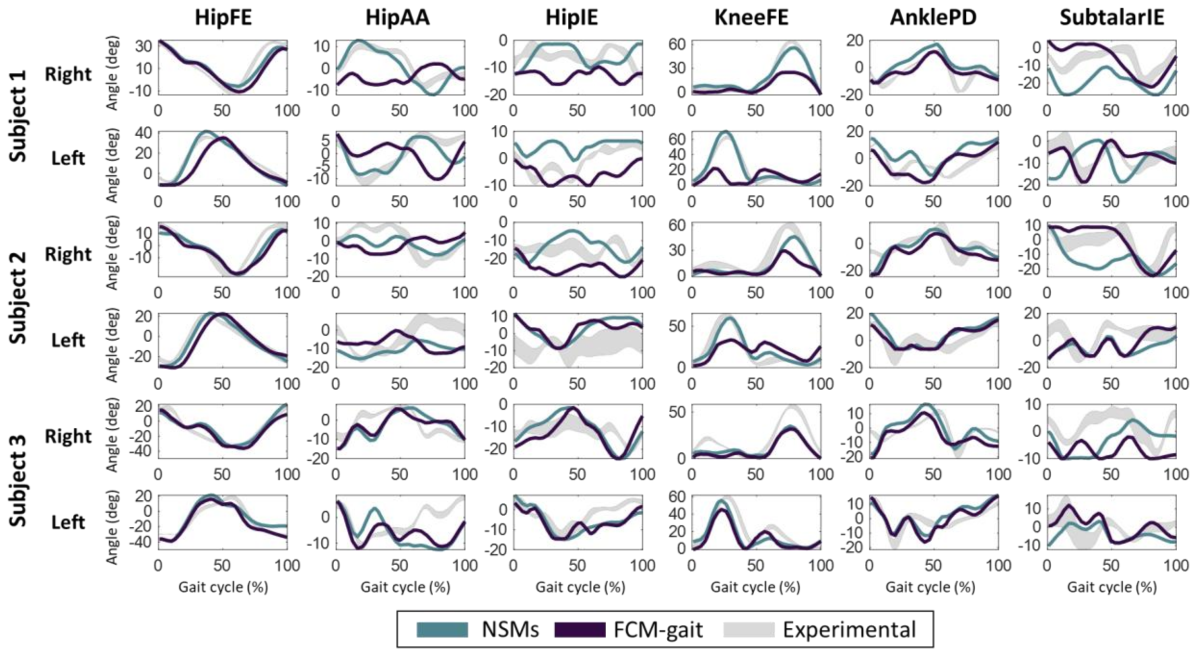

Fig S14. The predicted hipFE, hipAA, hipIE, kneeFE, anklePD and subtalarIE angles of NSMs and FCM-gait, compared to the respective experimental joint angles for the three subjects.

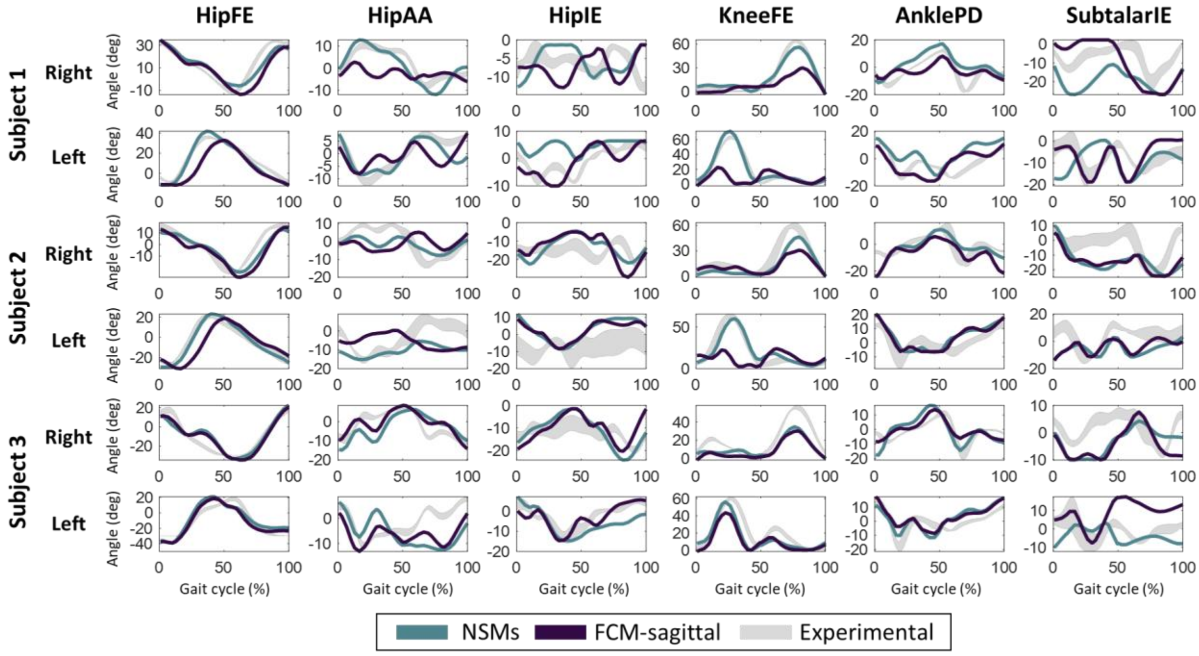

Fig S15. The predicted hipFE, hipAA, hipIE, kneeFE, anklePD and subtalarIE angles of NSMs and FCM-sagittal, compared to the respective experimental joint angles for the three subjects.

References

[1] Bhargava LJ, Pandy MG, Anderson FC. A phenomenological model for estimating metabolic energy consumption in muscle contraction. Journal of biomechanics. 2004;37(1):81–88.
[2] Jackson JN, Hass CJ, Fregly BJ. Development of a subject-specific foot-ground contact model for walking. Journal of biomechanical engineering. 2016;138(9):091002.
[3] Van Den Bogert AJ, Blana D, Heinrich D. Implicit methods for efficient musculoskeletal simulation and optimal control. Procedia Iutam. 2011;2:297–316.
